# Typhoon storm surge in the southeast Chinese mainland modulated by ENSO

**DOI:** 10.1038/s41598-021-89507-7

**Published:** 2021-05-12

**Authors:** Xingru Feng, Mingjie Li, Yuanlong Li, Fujiang Yu, Dezhou Yang, Guandong Gao, Lingjing Xu, Baoshu Yin

**Affiliations:** 1 CAS Key Laboratory of Ocean Circulation and Waves, Institute of Oceanology, Chinese Academy of Sciences, Qingdao, 266071 China; 2 Pilot National Laboratory for Marine Science and Technology, Qingdao, 266071 China; 3 Center for Ocean Mega-Science, Chinese Academy of Sciences, 7 Nanhai Road, Qingdao, 266071 China; 4 University of Chinese Academy of Sciences, Beijing, 100029 China; 5 National Marine Environmental Forecasting Center, Beijing, 100081 China; 6 CAS Engineering Laboratory for Marine Ranching, Institute of Oceanology, Chinese Academy of Sciences, Qingdao, 266071 China

**Keywords:** Physical oceanography, Natural hazards

## Abstract

In the past decade (2010–2019), the annual maximum typhoon storm surge (AMTSS) accounted for 46.6% of the total direct economic loss caused by marine disasters in Chinese mainland, but its prediction in advance is challenging. By analyzing records of 23 tide-gauge stations, we found that the AMTSSs in Shanghai, Zhejiang and Fujian show significant positive correlations with the El Niño-Southern Oscillation (ENSO). For the 1987–2016 period, the maximum correlation is achieved at Pingtan station, where correlation coefficient between the AMTSS and Niño-3.4 is 0.55. The AMTSS occurring in El Niño years are stronger than those in non-El Niño years by 9–35 cm in these areas. Further analysis suggests that a developing El Niño can greatly modulate the behaviors of Northwest Pacific typhoons. Strong typhoons tend to make landfall in southeast China with stronger intensities and northward shifted landfall positions. This study indicates that the modulation effect by ENSO may provide potential predictability for the AMTSS, which is useful for the early alert and reduction of storm surge damages.

## Introduction

The Northwest Pacific is the home to about one third of the tropical cyclones^1,2^ occurring over the globe. Passages of typhoons over coasts give rise to storm surges which constitute a major type of marine disasters in China (Bulletin of China Marine Disaster; http://www.mnr.gov.cn/sj/sjfw/hy/gbgg/zghyzhgb/). In 2019, for example, various types of marine disasters in China led to a total direct economic loss of 11.703 billion CNY, of which 11.638 billion CNY was by typhoon storm surges. Particularly, the storm surge caused by typhoon Lekima, the annual maximum typhoon storm surge (AMTSS) event of 2019, contributed by as much as 10.288 billion CNY, accounting for the majority of the total economic loss by all marine disasters. The total direct economic loss brought by marine disasters over the past decade (2010–2019) was 100.122 billion CNY, 46.6% of which was caused by the AMTSS events. Therefore, the local AMTSS largely represents the trial of marine disasters for coastal facilities and alert/prevention strategies. To reduce its damage, prediction with a long leading time is of paramount need to support the early warning and mitigation activities. However, the prediction of storm surge is a challenging task, given the complexities in typhoon behaviors and dynamical responses of coastal ocean to typhoons^3,4^. With these regards, the deterministic portion of the AMTSS variability associated with low-frequency climate modes, such as El Niño-Southern Oscillation (ENSO), represents potential predictability for the AMTSS and is worthy of in-depth investigation.

ENSO is the most influential interannual climate variability mode over the tropical Pacific and has significant impacts on typhoon activities in the Northwest Pacific, modulating frequency, intensity, and trajectory of typhoons in a dramatic manner^2,5^. In El Niño years, the genesis area of typhoons migrates southeastward, rendering typhoons with a lengthened northwestward propagation pathway and more growth time prior to the landfall. Therefore, the overall typhoon landfall intensity tends to be stronger in El Niño years^5–7^ and exerts stronger influences in East Asian countries^8^. Wang and Liu^9^ suggested that the relationship between ENSO and typhoon activity is tight in the warm phase of the Pacific Decadal Oscillation (PDO) and degraded in the cold phase. Zhao and Wang^2^ showed that the influence of ENSO on typhoon frequency has become significantly stronger since 1998 than before, owing to increasing La Niña and central Pacific-type El Niño events.

Given the notable modulation effect on the Northwest Pacific typhoons by ENSO, we pose a hypothesis that the AMTSS in southeast China may exhibit interannual variability associated with ENSO, although influences of topography and coastline geometry have also been suggested to be essential^3,10,11^. The first step to test this hypothesis is to examine the statistical relationship between ENSO and storm surge using reliable observational records. For example, Colle et al.^12^ analyzed the storm surge events of New York City from 1959 to 2007 and detected dramatically more minor-surge events (0.6–1.0 m) under El Niño condition than La Niña condition. Based on model simulation results, Oey and Chou^13^ failed to identify an evident relationship between ENSO and the AMTSS along the coasts of Northwest Pacific. Feng et al.^14^ showed that the duration time of strong typhoon storm surge events (> 1 m) in some areas of China is closely associated with ENSO. In addition, there has been a huge body of literature reporting the strong forcing effects of ENSO on sea-level variabilities of the western tropical Pacific^15–20^, the South China Sea^21–23^, and the East China Sea^24^.

In spite of existing studies reviewed above, the relationship between the storm surge of southeast China and ENSO has not been clarified. A particular investigation is required to achieve reliable statistical results and gain insights into the underlying physical processes. This study utilizes the typhoon storm surge records from 23 tide-gauge stations located in the typhoon-prone areas of the southeast Chinese mainland to examine the relationship between the AMTSS with ENSO, and the underlying causes for the correlations are also explored. Studies on the impacts of ENSO on coastal sea levels are useful for strategy-making to mitigate the sea-level change consequences^25^. This is particularly true for the AMTSS that can directly cause disastrous damages to people residing in islands and coastal regions.

## Results

### Correlations between the AMTSS and ENSO

**Figure 1:**
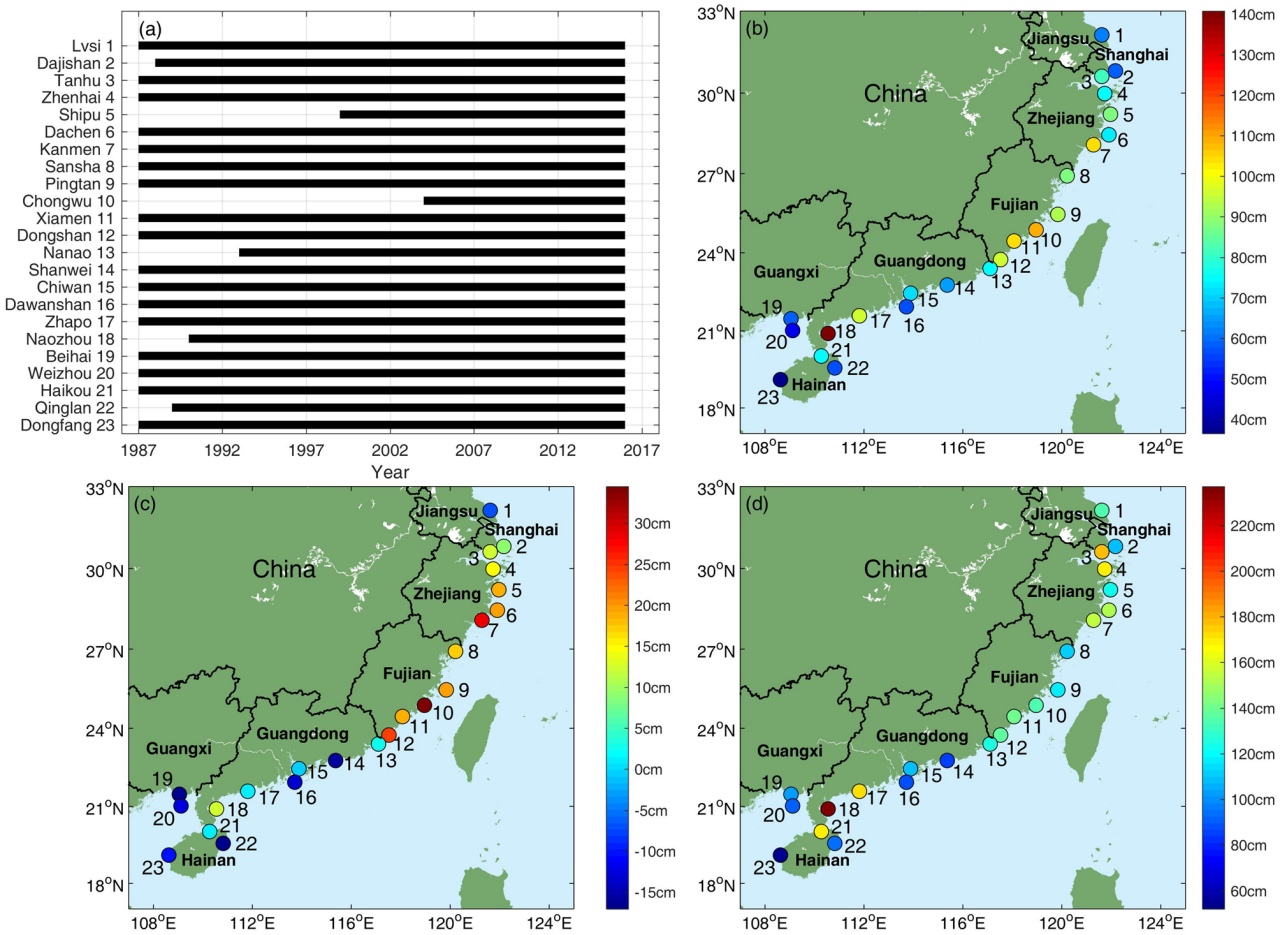
(**a**) Data duration of tide-gauge stations. (**b**) The average AMTSS in El Niño years. (**c**) Difference in the average AMTSS between El Niño and non-El Niño years. (**d**) The maximum values of AMTSS in El Niño years. Figures were plotted using MATLAB R2018b (http://www.mathworks.com/).

The hourly water-level data from 23 tide-gauge stations along the southeast Chinese mainland coast (Fig. 1a) are used to calculate the storm surge and the AMTSS (see “Methods”). To quantify the impacts of ENSO on the AMTSS, the spatial distributions of composite AMTSS for El Niño years (10 years) are shown in Figs. 1b, along with its difference from that of non-El Niño years (20 years) shown in Fig. 1c. See “Methods” for the definitions of El Niño and non-El Niño years. During El Niño years (Fig. 1b), most of the AMTSSs in the Zhejiang and Fujian provinces exceed 70 cm, and those in Guangdong, Guangxi, and Hainan are relatively weaker except at Naozhou station of Guangdong province (Station 18). Figure 1c suggests that the AMTSSs in Shanghai, Zhejiang, and Fujian are enhanced by 9 ~ 35 cm in El Niño years, as compared with those in non-El Niño years. The largest enhancement is seen in Chongwu, Fujian province, where the difference reaches 35 cm. The differences in Kanmen and Dongshan are also large, both exceeding 24 cm. In some stations of Jiangsu, Guangdong, Guangxi, and Hainan provinces, the AMTSSs in El Niño years are weaker than those in non-El Niño years. Among others, the differences in Shanwei, Beihai, Qinglan, and Dongfang are from − 17 to − 10 cm, indicating an overall suppression effect of El Niño on the local AMTSS.

We further compute the maximum AMTSS for El Niño years at each station (Fig. 1d). All the stations in Shanghai, Zhejiang, and Fujian have observed strong surges of > 100 cm, which can cause storm surge disasters when superimposed on high tidal levels. It is also noticeable that at Zhapo and Naozhou stations of Guangdong and Haikou station of Hainan, the maximal AMTSSs are even stronger. These high AMTSSs are not enhanced by El Niño years. For example, the maximum surge for El Niño year was 237 cm occurred in 2003, but in the non-El Niño year of 2014 the maximal surge reached 428 cm. The strong surges in these three stations may be due to local topography and the coastline geometry, which are favorable for the water accumulation under wind forcing^13,26^. This effect can be seen in Fig. 5f during Typhoon Mujigae in 2015.

**Figure 2:**
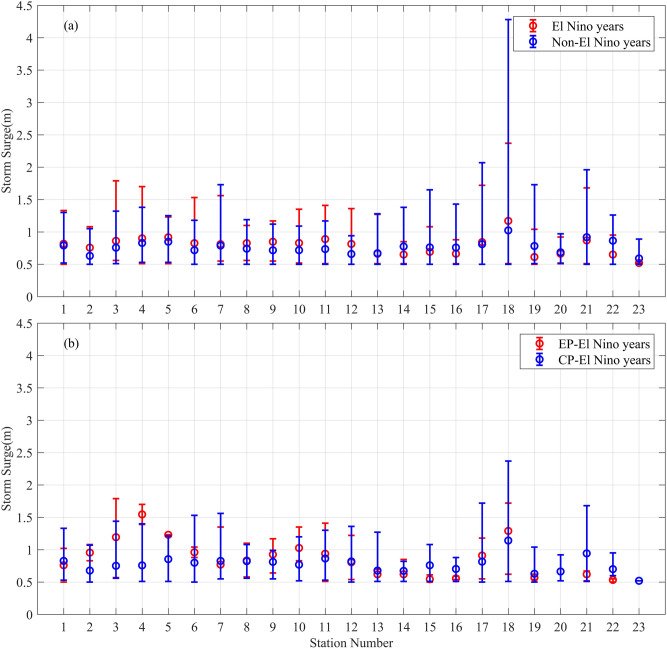
(**a**) Distribution of all typhoon storm surge ≥ 0.5 m at each station during certain El Niño (red) and non-El Niño (blue) years. (**b**) Same as (**a**) but for EP-El Niño (red) and CP-El Niño (blue) years. Note that there were no typhoon storm surge ≥ 0.5 m occurred during EP-El Niño years at Stations 20 and 23. The circle denotes the mean value, and the bar denotes the value range of all surges. Figures were plotted using MATLAB R2018b (http://www.mathworks.com/).

Besides the AMTSS, we also analyzed the distributions of surges at each station based on all the typhoons, and the results are shown in Fig. 2. Figure 2a shows that the mean values of typhoon storm surges at the stations north of Dongshan (in Fujian, Zhejiang and Shanghai) are larger in El Niño years than those in non-El Niño years by 2.6–15.5 cm, which is also true for the maximum storm surge at most of these stations. For stations south of Nanao (in Guangdong, Guangxi and Hainan), all the maximum storm surges are weaker in El Niño years than that in non-El Niño years by 1 ~ 191 cm. These results confirm the conclusion that the El Niño events can actually enhance the storm surges in Shanghai, Zhejiang, and Fujian, while impose an overall weakening effect on the storm surges in Guangdong, Guangxi and Hainan.

**Figure 3:**
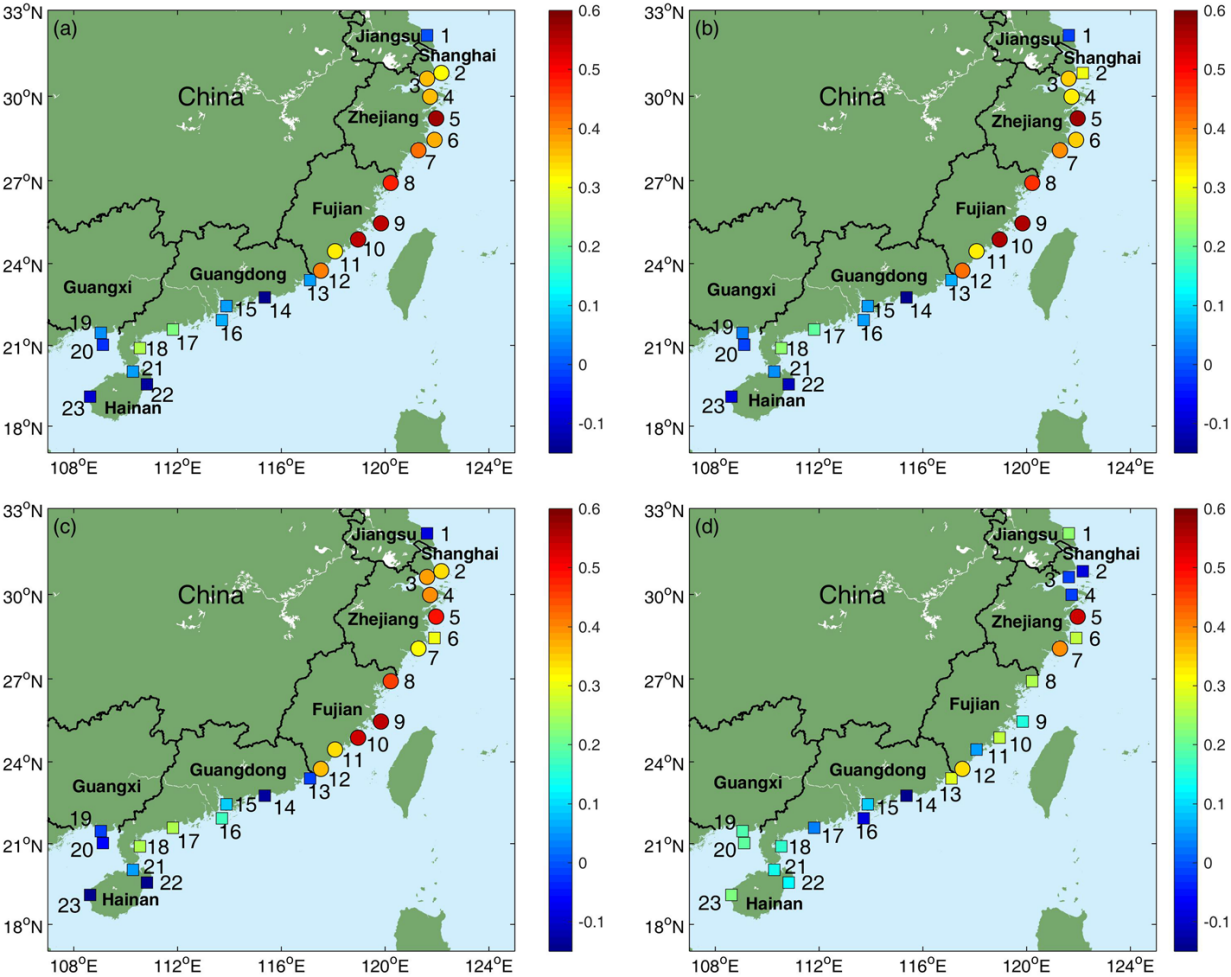
Correlation coefficients between the annual maximum storm surges (AMTSSs) and (**a**) Niño-3.4 index, (**b**) ONI index, (**c**) Niño 3 index and (**d**) EMI index at different tide-gauge stations. Circles and squares represent correlations significant and insignificant at 90% confidence level, respectively. Figures were plotted using MATLAB R2018b (http://www.mathworks.com/).

**Figure 4:**
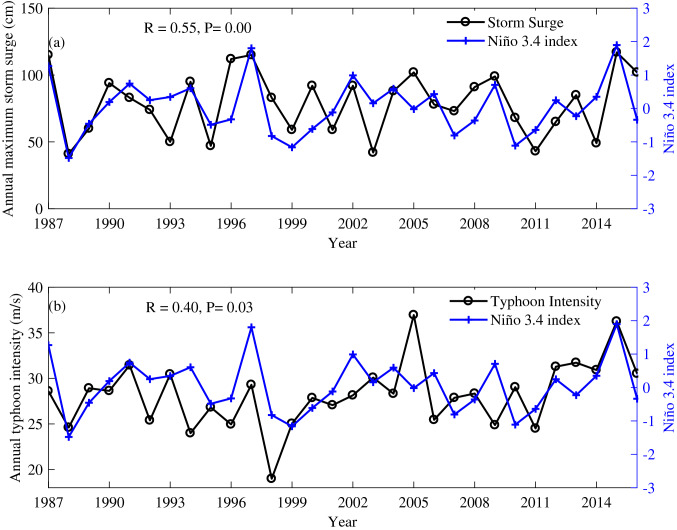
Time series of (**a**) the AMTSS at Pingtan and (**b**) the annual-mean typhoon intensity at its landfall, compared with Niño-3.4 index averaged over May–November.

To investigate how the ENSO modulates the AMTSSs along the coasts of southeast Chinese mainland, their linear correlation coefficients with Niño-3.4, ONI (Oceanic Niño Index), Niño-3 and EMI (El Niño Modoki Index) indexes are computed (Fig. 3). It can be seen that the AMTSS’s correlations with Niño-3.4 are quite close to those with ONI (Fig. 3a,b). The correlation coefficient between ONI and Niño-3.4 during 1987 to 2016 is 0.9954, and therefore similar conclusions can be reached regardless the choice between ONI and Niño-3.4. Therefore, in the following, we mainly adopt Niño-3.4 to represent ENSO. Figure 3a shows that the AMTSS at the tide-gauge stations in Shanghai, Zhejiang, and Fujian all show significant (> 90% confidence level, assuming each annual sample containing independent degree of freedom) positive correlations with the Niño-3.4 index. The highest correlation occurs at Pingtan station of Fujian province, reaching *r* = 0.55 during 1987–2016 (Figs. 3a, 4a). That is to say, the coastal areas of Shanghai, Zhejiang, and Fujian tend to suffer from stronger AMTSS in El Niño years. Taking Pingtan station as an example, during the two super El Niño events (1997 and 2015), the AMTSSs are 115 cm and 117 cm respectively, significantly greater than those in non-El Niño years (Fig. 4a). In the Guangdong, Guangxi, and Hainan provinces, the correlations between the AMTSS and ENSO are insignificant. It is interesting to note that there are negative correlations seen in several stations, such as Shanwei, Weizhou, Qinglan, and Dongfang, suggesting overall weaker AMTSSs in El Niño years.

Figure 3c and d show impacts of eastern Pacific (EP) type and central Pacific (CP) type El Niño events, quantified by Niño-3 and EMI respectively, on storm surge. The AMTSS’s correlations with Niño-3 show very similar distribution to those with Niño-3.4, except for Dachen (Station 6 in Zhejiang province) where the correlation with Niño-3 is insignificant (*r* = 0.30, *p* = 0.11). While for the EMI index, significant correlations with AMTSS were only found at 3 stations, i.e., Shipu, Kanmen and Dongshan. This may be due to the fact that all the EP-El Niño events (1987, 1997 and 2015) were strong and exert robust impacts on AMTSSs. By contrast, CP-El Niño events during 1987–2016 were weaker and their impacts on AAMTSs are limited, leading to degraded correlations between EMI and AMTSS. This result may be affected by data length which does not cover sufficient number of El Niño and surge events (Fig. 1a).

For further confirmation, we also compared the distributions of surges (≥ 0.5 m) based on all the typhoons during El Niño and CP-El Niño years, and the results are shown in Fig. 2b. It can be seen that at the stations north of Dongshan (in Fujian, Zhejiang and Shanghai), the mean values of typhoon storm surges in the EP-El Niño years are larger or close to those in CP-El Niño years. By contrast, at the stations south of Nanao (Guangdong, Guangxi and Hainan), almost all of the maximum storm surges are weaker in the EP-El Niño years than that in CP-El Niño years. These results indicate that the ENSO’s effect on the storm surge is stronger for EP-El Niño events than for CP-El Niño events in the present study period. Studies based on extended storm surge observation and model simulations will be helpful in the future to clarify the impacts of ENSO diversity on storm surge.

Figure 3 indicates that Niño-3.4 may be a good representation of ENSO variability and useful for the prediction of AMTSS in southeast China. The analysis presented above overall suggests a significant enhancement of AMTSS in Shanghai, Zhejiang, and Fujian by El Niño events and a weaker suppressing effect in Guangdong, Guangxi, and Hainan. Given the potential importance for storm surge prediction, this modulation effect by ENSO and its well-organized spatial distribution are worthy of in-depth understanding.

### Effects of typhoon variability

**Figure 5:**
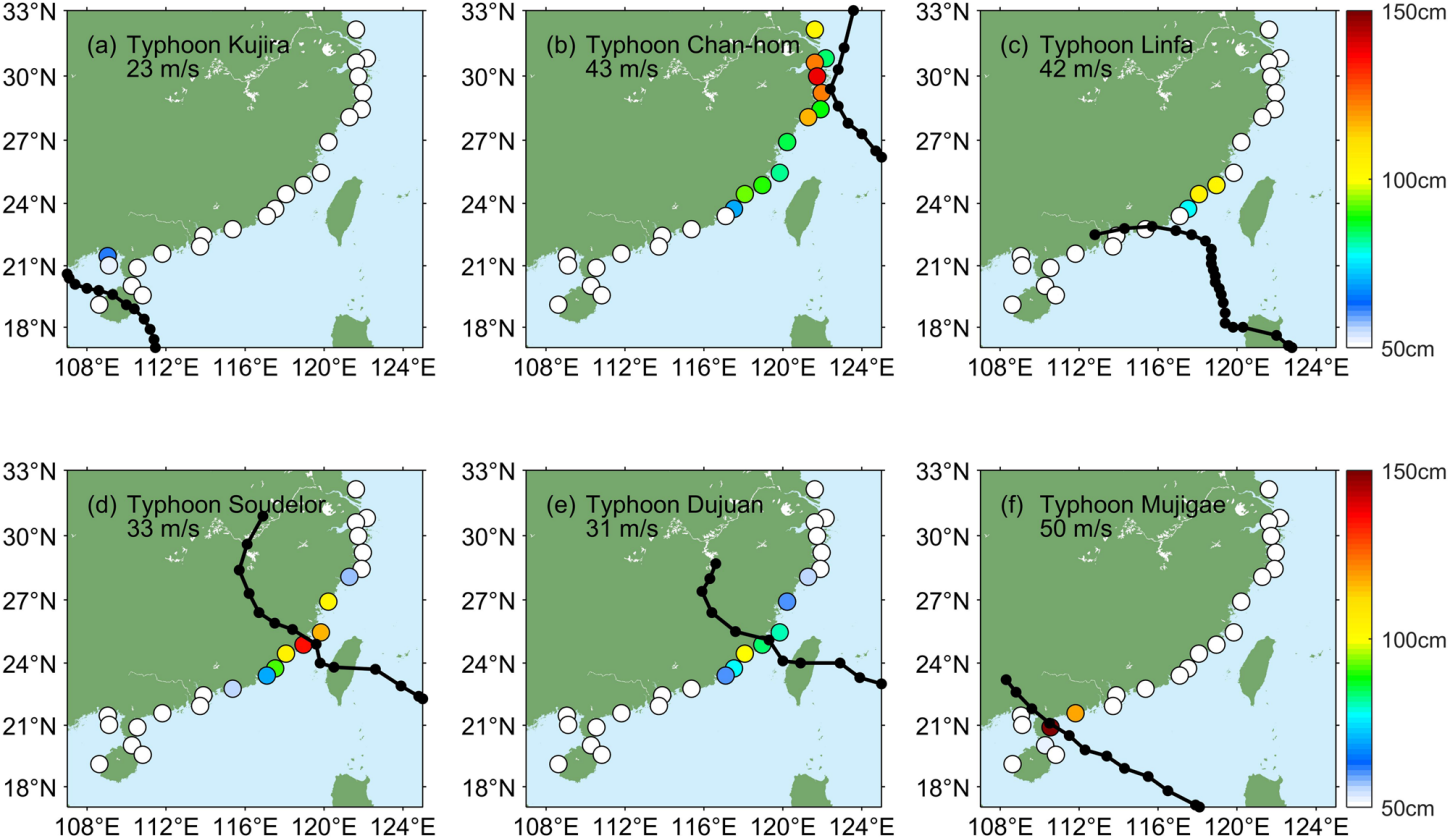
Observed maximum storm surge induced by the typhoons of (**a**) Kujira, (**b**) Chan-hom, (**c**) Linfa, (**d**) Soudelor, (**e**) Dujuan and (**f**) Mujigae in 2015. Surge values below 50 cm are plotted white. The maximum wind speed of the typhoon landfall is specified in each panel. Figures were plotted using MATLAB R2018b (http://www.mathworks.com/).

Next, we attempt to explain the observed correlations between AMTSS and ENSO through changes in typhoon behaviors. Previous studies have reported the strong interannual variability in intensity of typhoons formed in the Northwest Pacific^5–7^ which caused most of large storm surges in Chinese mainland at landfall (Bulletin of China Marine Disaster). The observed annual-mean landfall intensity of typhoons shows a correlation of r = 0.40 with the Niño-3.4 index (Fig. 4b). Such ENSO-related typhoon variability may serve as a linkage between AMTSS and ENSO. However, as shown in Fig. 3a, the correlation between the AMTSS and ENSO shows evident spatial variation among different stations. We attempt to explain this variation by exploring the ENSO’s impact on the landfall positions of typhoons. As an example, for the effect of typhoon landing position on the storm surge distribution, we first look at 6 typhoon events occurred in 2015. Their trajectories and the corresponding maximum storm surges observed by tide-gauge stations were shown in Fig. 5. One can see that the large storm surges (≥ 50 cm) mainly appeared near the landing positions of the typhoon, and stations on the right-hand-side of the typhoon trajectory generally observed greater surges, consistent with the conventional knowledges established by previous studies^26^. The typical case of 2015 indicates that El Niño events may enhance the AMTSSs along Shanghai, Zhejiang, and Fujian coasts by causing strong typhoons with higher-latitude landing positions.

**Figure 6:**
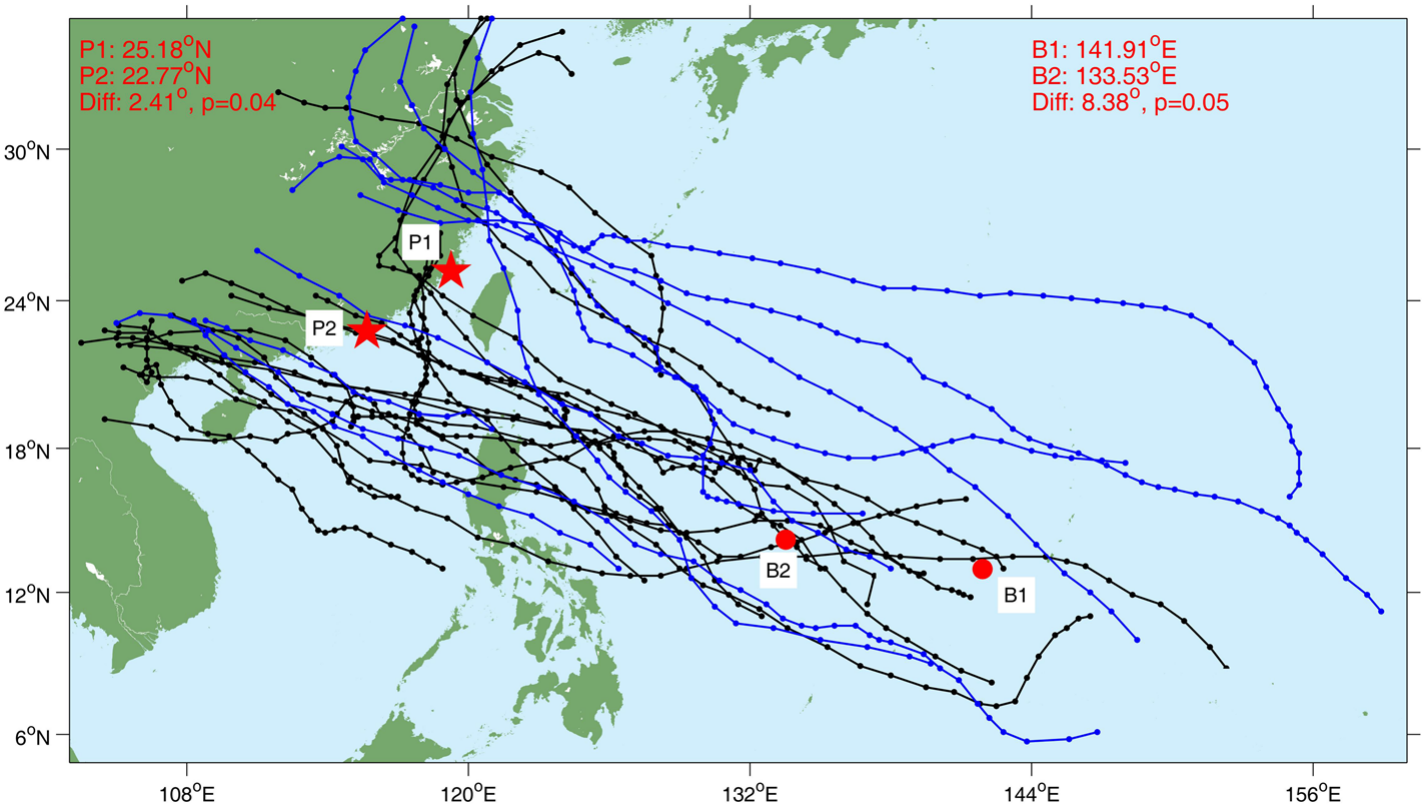
Typhoon paths of the annual strongest typhoons in El Niño (blue) and non-El Niño (black) years during 1987–2016. B1 and B2 (P1 and P2) represent the average genesis (landfall) positions of these typhoons in El Niño and non-El Niño years, respectively. Figures were plotted using MATLAB R2018b (http://www.mathworks.com/).

Then, we compare the paths of the annual-strongest typhoons in El Niño and non-El Niño years (Fig. 6). In El Niño years, there were more strong typhoons generated in the eastern portion of the Philippine Sea (e.g., east of 140°E), while most non-El Niño years had the strongest typhoons generated in the South China Sea and near the Philippine coast. The average longitude of typhoon genesis is 141.91°E for El Niño years versus 133.53°E for non-El Niño years. The difference of 8.38° is significant at 95% confidence level. Given that the Northwest Pacific typhoons generally travel northwestward, the El Niño-year typhoons tend to have a lengthened pathway over warm ocean before transected by landmass and therefore achieve more time for growth^27,28^. As such, these typhoons can make landfall at higher-latitude regions in Chinese mainland such as Zhejiang and Fujian and attain stronger landfall intensity. This explains why the AMTSSs in Shanghai, Zhejiang, and Fujian bear significant positive correlations with ENSO and tend to be enhanced in El Niño years.

To confirm the impact of ENSO on the typhoon landing position, we contrast the typhoon landing positions for El Niño years and non-El Niño years. The average typhoon landing latitude of the annual-strongest typhoons was 25.18°N for El Niño years and 22.77°N for non-El Niño years, and the difference exceeds 95% confidence. For further confirmation, more analysis was performed for the landfall locations of the typhoons. As for the significance test, the Students’ t-test was performed. Considering the small sample size, we also adopted the Wilcoxon–Mann–Whitney test^29,30^ to confirm the significance of the difference. The two methods obtained similar results (Table 1).

**Table 1:** Latitude of typhoon landfalling locations in the southeast Chinese mainland.

Wind speed when made landfall	1965–2016				1987–2016			
	Difference significance (p value)		Mean landfall latitude		Difference significance (p value)		Mean landfall latitude	
	Student’s t test	Wilcoxon–Mann–Whitney test	El Niño years	non-El Niño years	Student’s t test	Wilcoxon–Mann–Whitney test	El Niño years	non-El Niño years
All	0.21	0.18	22.77	22.40	0.74	0.47	22.83	22.68
≥ 15 m/s	0.45	0.28	22.97	22.74	0.92	0.37	22.97	23.02
≥ 20 m/s	0.49	0.25	23.11	22.85	0.99	0.42	23.12	23.11
≥ 25 m/s	0.20	0.10	23.17	22.58	0.26	0.13	23.59	22.87
≥ 30 m/s	0.04	0.04	23.73	22.48	0.06	0.05	24.23	22.68
≥ 35 m/s	0.01	0.01	24.34	22.33	0.08	0.05	24.69	22.90
strongest	0.03	0.03	24.75	22.73	0.04	0.05	25.18	22.77

According to Table 1, there were generally more northern typhoon landfalls in El Niño years than in non-El Niño years during 1987–2016. The average typhoon landing latitude of all typhoons was 22.83°N for El Niño years and 22.68°N for non-El Niño years, although the difference is below 90% significance. For the strong typhoons defined with a landfall wind speed criterion of > 30 m s^−1^, the average landfall location is significantly more northward in El Niño years, and their average landing latitude was 24.23°N and 22.68°N for El Niño and non-El Niño years, respectively, with the difference significant at 95% confidence level. If we extend the analysis to 1965–2016 which is the satellite era for typhoon observation, similar results were obtained. These comparisons indicate that the landfall locations of strong typhoons tend to be more northward during El Niño years than in non-El Niño years.

Existing studies have pointed out that the typhoons tend to impact higher latitudes during El Niño years due to the eastward shift of the typhoon formation area and the influence of the subtropical high^28,31^, which is consistent with our results. Then, a question arises as for why such impact is more evident for the strong typhoons? One possible reason is that there is a northward meridional deflection when the typhoon moves westward in the WNP due to the *ß*-effect^32^, and such meridional drift was more significant for the strong typhoons. That is to say, under the same background steering flow, it is easier for the strong typhoons to keep its direction northwestward than the weak typhoons. As such, strong typhoons in Niño years tend to have more northward landfall locations than those formed in non-Niño years. This notion is supported by Fig. 6, which shows that the majority of the annual-maximum typhoons move northwestward. Liu and Chan^33,34^ showed that, landfalling typhoons in the South China (Guangdong and Hainan provinces) tend to be normal or below normal during El Niño years. Therefore, there are more strong typhoons making landfalls in Guangdong, Guangxi, and Hainan in non-El Niño years, which can enhance local AMTSSs therein. This may partly explain the prevailing negative correlations seen in these areas (Fig. 3a).

We also contrasted the characteristics of strong typhoons with northern and southern landfall positions, dubbed as northern-landfall and southern-landfall typhoons (north of P1 and south of P2 in Fig. 6), respectively. The landfall intensity of northern-landfall typhoons shows a correlation of r = 0.35 with Niño-3.4 index, significant at 94% confidence level. This correlation is insignificant (r = 0.10) for southern-landfall typhoons. One possible explanation for this difference is that the southern-landfall typhoons are mostly formed in the South China Sea or near the Philippine coasts (mean formation position 130.91°E, 13.50°N), while the northern-landfall typhoons are mostly formed in the open ocean region of the Northwest Pacific (mean formation position 141.02°E, 15.80°N). As such, ENSO exert stronger modulation effect on the northern typhoons during its formation and propagation. The southern typhoons might be also affected by other local ocean or atmospheric processes, and therefore show a lower correlation with ENSO. The variability of typhoon behaviors and storm surges in this region are more complicated than those in the north and demands further investigation.

## Conclusions and discussion

The typhoon storm surge is a major type of marine disasters in China causing serious life and economical losses every year, but its prediction with a long leading time is difficult. The possible modulation effect on AMTSS by ENSO was investigated based on the observational records at 23 tide-gauge stations located in the typhoon-prone coastal areas of the southeast Chinese mainland. The results reveal a well-structured correlation pattern over the southeast China coasts. The AMTSSs along the coasts of Shanghai, Zhejiang, and Fujian show significant correlations with Niño-3.4 index. For all the stations with the full-length records of 1987–2016, the maximum correlation coefficient of r = 0.55 is found at Pingtan, Fujian province. The AMTSSs occurring in El Niño years are stronger than those in non-El Niño years by 9–35 cm in these areas. Correlations in Guangdong, Guangxi, and Hainan are generally low and insignificant, among which negative correlations are seen at Shanwei, Weizhou, Qinglan, and Dongfang.

These relationships are largely explained by the impact of ENSO on the intensities and landing positions of the Northwest Pacific typhoons that make landfalls in southeast China. Strong typhoons (defined with a landfall wind speed criterion of > 30 m s^−1^) in El Niño years tend to originate from the eastern portion of the Philippine Sea and therefore have a lengthened path over the warm ocean and more time to growth. As such, their landing positions are shifted northward (24.23°N on average), and their landing intensities tend to be enhanced, as compared with typhoons in non-El Niño years (22.68°N on average in landing position). These typhoon events cause stronger AMTSSs along Shanghai, Zhejiang, and Fujian coasts in El Niño years. By contrast, the majority of strong typhoons that made landfall in Guangdong, Guangxi, and Hainan, were generated in the South China Sea or near the Philippine coasts, thus they receive limited modulation effect by ENSO along their shortened propagation pathway toward China. As such, the correlations between the AMTSS and ENSO in these provinces are largely insignificant. In addition, Fig. 5 suggests that the typhoons with a southern landing position tend to influence a smaller area nearby than those landing in the north, and this may be a reason why the AMTSS/ENSO correlations in Guangdong, Guangxi, and Hainan provinces are mostly insignificant. But such impact is proved to be statistically insignificant by comparing the average number of the stations that observed storm surges of ≥ 50 cm, for the strong typhoons that made landfall north of position P1 and south of P2 (P1 and P2 are shown in Fig. 6). We also analyzed the distributions of surges at each station based on all the typhoons, and the results achieved similar conclusion with that based on AMTSSs.

**Figure 7:**
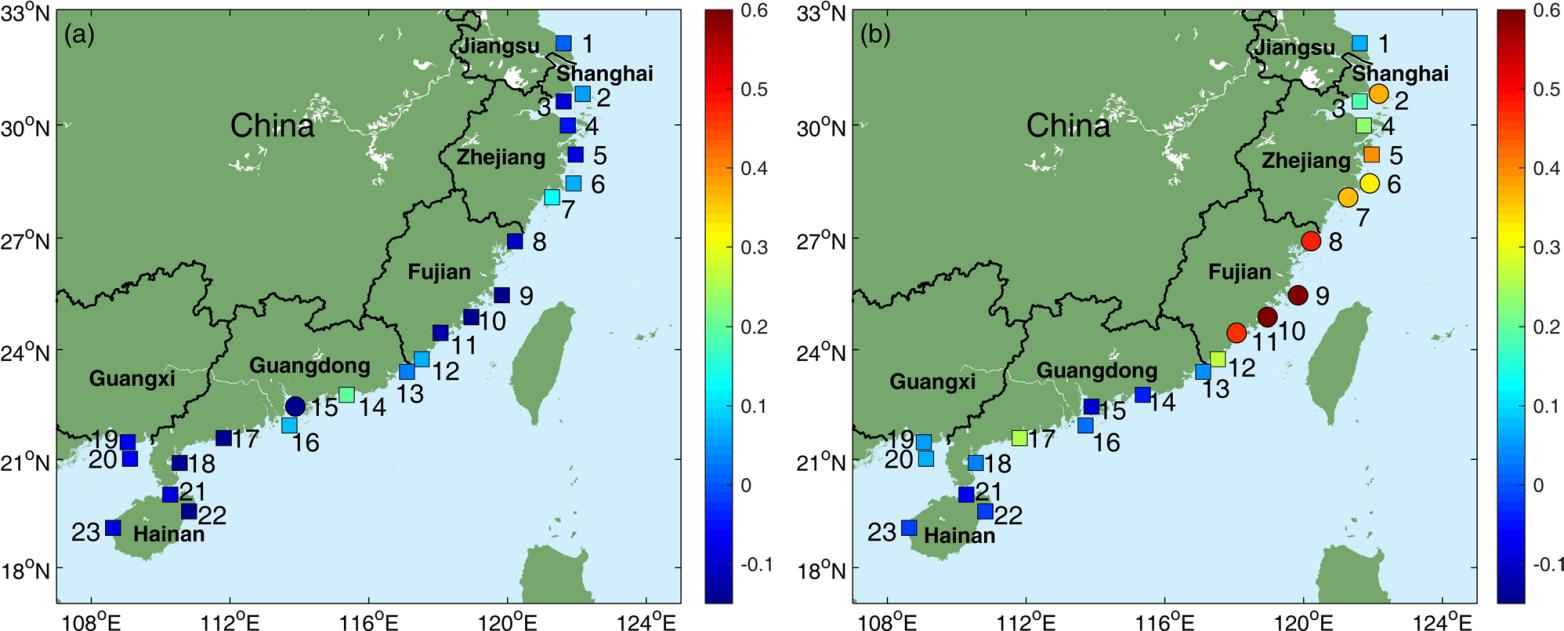
Correlation coefficients between the Niño-3.4 index and annual number of (**a**) Minor-surge events and (**b**) Major-surge events. Figures were plotted using MATLAB R2018b (http://www.mathworks.com/).

Our results show that there are generally more northward landfalls in El Niño years for all the WNP typhoons, but the difference is insignificant in statistical sense. Yet, the difference for strong typhoons is significant. To gain further insights, following Colle et al.^12^, we divided the storm surges into minor-surge (0.5–1.0 m) and major-surge (> 1 m) events. The correlation coefficients between Niño-3.4 index and the annual numbers of the two types of surge events were provided in Fig. 7a and b. There is no significant correlation between the minor-surge and ENSO events, while the major-surge events were significantly modulated by ENSO for the coasts of Fujian, Zhejiang and Shanghai. Based on these results, we can conclude that only strong typhoon storm surge events were significantly modulated by ENSO for the northern part of southeast Chinese mainland. This is also consistent with the conclusion that only the strong typhoons’ landfalling locations were significantly affected by ENSO.

This study postulates ENSO as a remote forcing regime for the AMTSS in the typhoon-prone coastal areas of Chinese mainland. Since useful prediction for ENSO can be achieved by several months ahead by the present generation of forecast systems, this can be rather useful for enhancing the probabilistic prediction of storm surges and provide a precious time window for warning, prevention, and mitigation steps. In other words, precursors of El Niño over the tropical Pacific may be used as predictors for strong AMTSSs in Shanghai, Zhejiang, and Fujian, and proper preparation can be carried out to reduce the losses caused by storm surges. We are also aware that ENSO is only one of the many processes modulating typhoons and storm surges. Extreme typhoons and storm surges can also take place in non-El Niño years (see Fig. 4).

This study describes the relationship between the AMTSS in southeast China and ENSO index and explores the underlying modulation processes. Yet both typhoon storm surge and ENSO are complex in dynamics, and more in-depth investigation is required to understand the impact of ENSO on China’s typhoon storm surges and incorporate into existing prediction systems. The AMTSS at Lvsi, Jiangsu province was not significantly affected by ENSO, although it locates near the high-correlation region (Fig. 3a). This may be due to the geometry of the coastline near Lvsi, which may be also true for the AMTSS of South China Sea. As previous studies have shown that the geometry can affect the surge responses significantly^3,26^. So, in future research, carefully designed numerical simulation studies are required to clarify the key mechanisms of storm surge response to ENSO-modulated typhoon events. In addition, in this study, different impacts of EP and CP type El Niño on the storm surge were explored, and the conclusions are in accordance with the previous studies that both EP and CP-El Niño events can modulate the typhoon intensities and tracks^36,39^. The possible impacts of ENSO diversity on the northwest Pacific typhoons^2^ and China storm surges are also worth further in-depth investigation to improve our understanding for the linkage between coastal marine disasters and climate change/variability.

## Methods

The hourly water-level data from 23 tide-gauge stations located at the typhoon-prone coastal areas of Chinese mainland was used to calculate the storm surge during typhoon processes. The spatial distribution of the tide-gauge stations and data duration are shown in Fig. 1. Data of 17 stations can fully cover the 30 years’ period of January 1987-December 2016, except for the Dajishan, Shipu, Chongwu, Nanao, Naozhou and Qinglan stations (Fig. 1a). The storm surge ${\upeta }_{\mathrm{surge}}$ is defined as.

1 $${\upeta }_{\mathrm{surge}}=\upeta -{\upeta }_{\mathrm{tide}}$$

where $\upeta$ is the observed water level, and ${\upeta }_{\mathrm{tide}}$ is the water level of the predicted tide. Then, we can get the AMTSS by finding the maximum ${\upeta }_{\mathrm{surge}}$ during all the typhoon events for each year and each station.

The typhoon data used in this study was obtained from the China Meteorological Administration-Shanghai Typhoon Institute (CMA_STI), which provides typhoon locations and intensities at 6-h intervals for the period of 1987–2016. The Niño-3.4 and ONI (Oceanic Niño Index) indexes were adopted to represent the overall ENSO variability and can be obtained from the NOAA (National Oceanic and Atmospheric Administration) Physical Sciences Laboratory. Niño-3.4 index (ONI index) is calculated by the average (3 month running mean) sea surface temperature (SST) anomaly over the NINO34 region (170°W–120°W, 5°S–5°N). As previous studies have shown that eastern Pacific (EP) and central Pacific (CP) type El Niño have different impacts on WNP typhoon activities such as the genesis locations and tracks^35,36^. So, to investigate such impacts on the storm surge, we adopted the Niño 3 and EMI (El Niño Modoki Index) indexes to represent the EP and CP type El Niño events respectively. Niño 3 index is calculated by the average SST anomaly over the region (150°W–90°W, 5°S–5°N). The SST data used for calculating Niño-3.4, ONI and Niño 3 was the Extended Reconstructed Sea Surface Temperature, Version 5^37^. The EMI index is calculated by the method of Ashok et al.^38^ and can be obtained at Japan Agency for Marine-Earth Science and Technology.

Correlation coefficients of the AMTSS and ENSO index were calculated at the 23 tide-gauge stations to quantify the covariance between the typhoon storm surge and ENSO. The average ENSO indexes for the extended typhoon season of May–November is used to represent the ENSO condition of a year and compute the correlations with typhoon intensity and AMTSS^2^. Both student’s t-test and Wilcoxon–Mann–Whitney test^29,30^ were used to test the statistical significance of the results. The identification of the EP-El Niño and CP-El Niño years for the period of 1987–2016 adopts the results of Zhao and Wang (2019). 1987, 1997 and 2015 are identified as EP-El Niño years, 1991, 1994, 2002, 2003, 2004, 2006 and 2009 are identified as CP-El Niño years.

## Data Availability

The Niño-3.4, ONI and Niño-3 indices were taken from NOAA Physical Sciences Laboratory (PSL, https://psl.noaa.gov/enso/dashboard.html); the EMI index was from Japan Agency for Marine-Earth Science and Technology (http://www.jamstec.go.jp/frsgc/research/d1/iod/modoki_home.html.en); the annual Bulletin of China Marine Disaster was from the Ministry of Natural Resources of the People’s Republic of China (http://www.mnr.gov.cn/sj/sjfw/hy/gbgg/zghyzhgb/); The typhoon data was from the China Meteorological Administration-Shanghai Typhoon Institute (http://tcdata.typhoon.org.cn/#/best-track/data-access). All the tide-gauge data used for our analysis are provided by the National Marine Data and Information Service and National Marine Environmental Forecasting Center and are available in Zenodo website (https://doi.org/10.5281/zenodo.3997307).
